# Insertion site and risk of peripheral intravenous catheter colonization and/or local infection: a post hoc analysis of the CLEAN 3 study including more than 800 catheters

**DOI:** 10.1186/s13756-024-01414-4

**Published:** 2024-06-05

**Authors:** Bertrand Drugeon, Nicolas Marjanovic, Matthieu Boisson, Niccolò Buetti, Olivier Mimoz, Jérémy Guenezan

**Affiliations:** 1 CHU de Poitiers, Service des Urgences Adultes –SAMU, 86-Centre 15-2, Rue de La Milétrie, Poitiers, Cedex 86021 France; 2 INSERM, U1070, Pharmacologie Des Agents Antimicrobiens Et Antibio-Résistances, Poitiers, France; 3 Alliance for Vascular Access Teaching and Research, Griffith University, Brisbane, QLD Australia; 4 INSERM CIC1402, CHU de Poitiers, Poitiers, France; 5 Université de Poitiers, UFR de Médecine Pharmacie, Poitiers, France; 6 Service d’Anesthésie Et Réanimations Et Médecine Péri-Opératoire, CHU de Poitiers, Poitiers, France; 7 Infection Control Program and WHO Collaborating Centre, Faculty of Medicine, Geneva University Hospitals, Geneva, Switzerland; 8 Université Paris-Cité, INSERM, IAME UMR 1137, 75018 Paris, France

**Keywords:** Peripheral intravenous catheter, Insertion site, Colonization, Infectious complication

## Abstract

**Aim:**

Although uncommon, infections associated with peripheral intravenous catheters (PIVCs) may be responsible for severe life-threatening complications and increase healthcare costs. Few data are available on the relationship between PIVC insertion site and risk of infectious complications.

**Methods:**

We performed a post hoc analysis of the CLEAN 3 database, a randomized 2 × 2 factorial study comparing two skin disinfection procedures (2% chlorhexidine-alcohol or 5% povidone iodine-alcohol) and two types of medical devices (innovative or standard) in 989 adults patients requiring PIVC insertion before admission to a medical ward. Insertion sites were grouped into five areas: hand, wrist, forearm, cubital fossa and upper arm. We evaluated the risk of risk of PIVC colonization (*i.e.*, tip culture eluate in broth showing at least one microorganism in a concentration of at least 1000 Colony Forming Units per mL) and/or local infection (*i.e.*, organisms growing from purulent discharge at PIVC insertion site with no evidence of associated bloodstream infection), and the risk of positive PIVC tip culture (*i.e.*, PIVC-tip culture eluate in broth showing at least one microorganism regardless of its amount) using multivariate Cox models.

**Results:**

Eight hundred twenty three PIVCs with known insertion site and sent to the laboratory for quantitative culture were included. After adjustment for confounding factors, PIVC insertion at the cubital fossa or wrist was associated with increased risk of PIVC colonization and/or local infection (HR [95% CI], 1.64 [0.92—2.93] and 2.11 [1.08—4.13]) and of positive PIVC tip culture (HR [95% CI], 1.49 [1.02—2.18] and 1.59 [0.98—2.59]).

**Conclusion:**

PIVC insertion at the wrist or cubital fossa should be avoided whenever possible to reduce the risk of catheter colonization and/or local infection and of positive PIVC tip culture.

**Supplementary Information:**

The online version contains supplementary material available at 10.1186/s13756-024-01414-4.

## Introduction

Peripheral intravenous catheters (PIVCs) are the most widely used medical devices in hospitals [1]. Every year, 2 billion PIVCs are sold worldwide [2]. Of these, 50% are subject to mechanical (accidental removal, dislodgment, leakage from insertion site, occlusion), vascular (phlebitis, diffusion) or infectious (local or bloodstream infection [BSI]) complications leading to PIVC failure [3]. PIVC failure is responsible for treatment interruptions which can be detrimental to patients. In addition, BSIs prolong hospitalization and increase treatment costs and mortality [4]. In a retrospective study conducted from January 2018 to March 2020, among the 9833 patients visiting our emergency department and hospitalized in a medical ward after insertion of a PIVC, 25 cases (0.2%) of PIVC-related BSI were identified. Of these, major complications occurred in nine patients (36%) including six deaths, one severe sepsis requiring intensive care unit admission, one thoracic spondylodiscitis, one mitral valve endocarditis and one deep pre-sacral abscess. Median additional hospital stay costs were estimated at €5,587 per case [5].

National guidelines have been developed to reduce the occurrence of these complications and to improve patient outcome. They include disinfecting hands with a hydro-alcoholic solution when handling the catheter or the line, preparing the skin with 2% chlorhexidine-alcohol, inserting the PIVC once the work area is dry using the no-touch technique, and applying a transparent film dressing over the PIVC insertion site.

The choice of insertion site to limit complications is still a matter of debate. Numerous studies have been conducted to identify risk factors for non-infectious complications. Overall, the upper extremities should be preferred to the lower limbs to reduce these complications, while avoiding the wrist and cubital fossa [6]. Little is known about the choice of PIVC insertion site to reduce the infectious risk. Therefore, we analyzed data collected during the CLEAN 3 trial to determine the risk of PIVC colonization and/or local infection and the risk of positive PIVC tip culture according to insertion site [7].

## Material and methods

CLEAN 3 was a randomized, 2 × 2 factorial clinical trial carried out at Poitiers University Hospital in France [7]. The trial has two main objectives: (1) to demonstrate the superiority of skin preparation with 2% chlorhexidine-alcohol over 5% povidone iodine-alcohol in preventing PIVC colonization, and (2) to demonstrate the superiority of a set of innovative devices including integrated PIVC, zero-reflux needless-connectors, disinfecting caps and single-use prefilled flush syringes over standard PIVC in extending the time elapsed between PIVC placement and PIVC failure. The investigators obtained written informed consent before study inclusion. The French Southwest and Overseas Ethics Committee and the French Drug Safety Agency approved the trial.

The trial enrolled adult patients (age ≥ 18 years) visiting the Emergency Department and requiring a single PIVC for a predictable duration of at least 48 h before being admitted to medical wards. Main exclusion criteria were known allergies to chlorhexidine or povidone iodine; suspicion of BSI at PIVC insertion; participation to another clinical trial aimed at reducing PIVC complications; skin injury at PIVC insertion site; PIVC placement in extremely urgently situation defined as patient triage score of 1 on Nurse Classification of Emergency Patients; suspicion of difficult PIVC insertion; and previous enrolment in the trial.

**Fig. 1:**
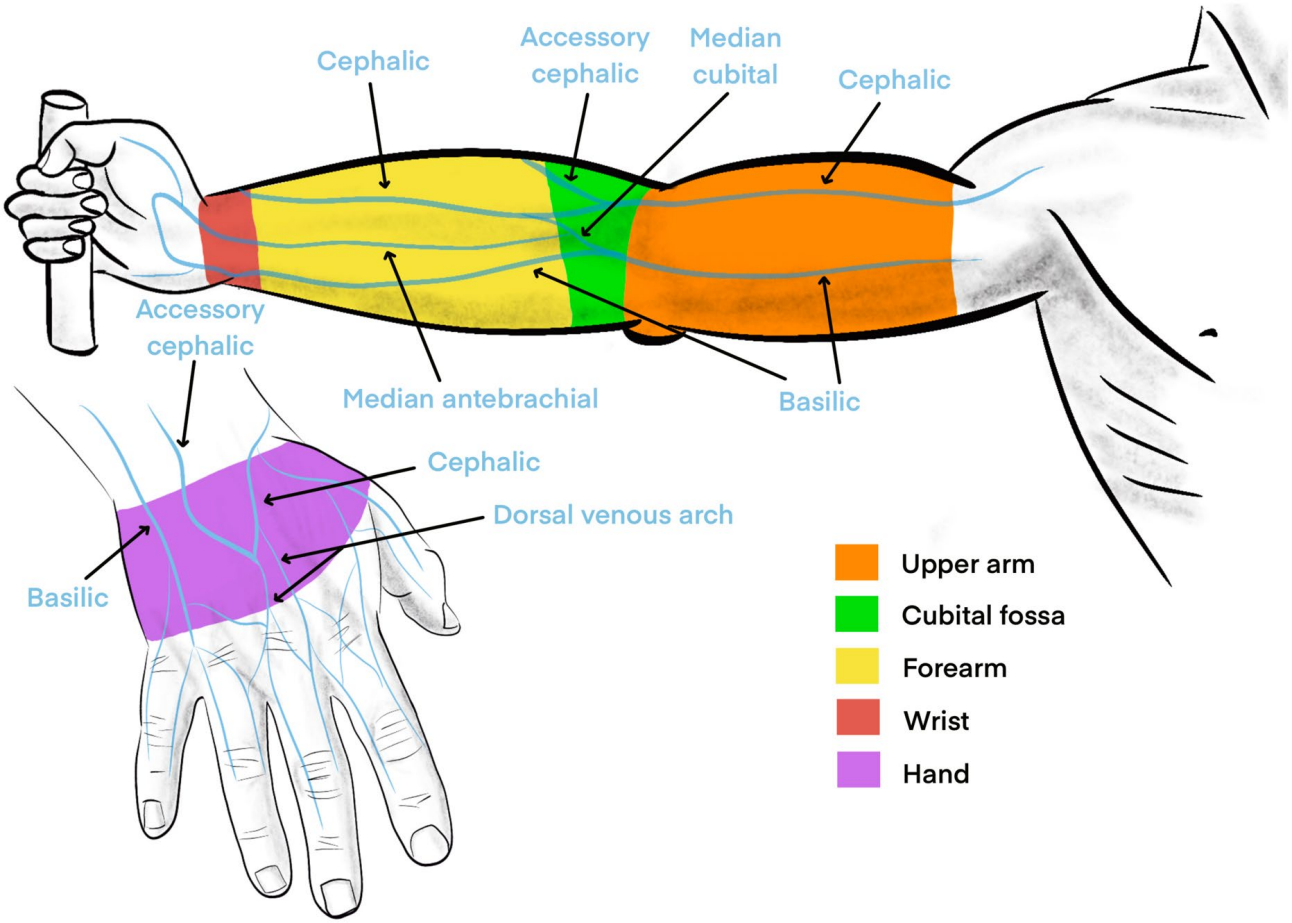
Peripheral venous catheter insertion sites were grouped into 5 areas. In blue, the names of the main veins

Patients were assigned to one of four groups according to the modalities of skin disinfection (2% chlorhexidine-alcohol or 5% povidone iodine-alcohol) and type of devices used (innovative or standard). PIVC were inserted and handled according to the French guidelines. PIVC insertion sites were selected according to the inserter and grouped into five areas (Fig. 1): hand, wrist, forearm, cubital fossa and upper arm. At PIVC removal, PIVC tips were sent to the main laboratory for quantitative culture.

*Catheter colonization* was defined as a PIVC-tip culture eluate in broth showing at least one microorganism in a concentration of at least 1000 colony forming units per mL (CFU/mL). *Local infection* was defined as organisms growing from purulent discharge at PIVC insertion site with no evidence of BSI. A *positive PIVC tip culture* was defined as a PIVC-tip culture eluate in broth showing at least one microorganism regardless of its amount. Characteristics of patients and PIVC, and risk factors for PIVC complications were collected prospectively by research staff.

### Statistical analysis

Characteristics of patients and PIVC were described as median (Interquartile range [IQR]) or number (proportion) as appropriate. First, we performed univariate analyses to identify associated covariates for PIVC colonization and/or local infection, and for positive PIVC tip culture. Then, we performed multivariate Cox models adjusted for covariates with *p* values < 0.20. Skin preparation (2% chlorhexidine-alcohol or 5% povidone iodine-alcohol) and type of devices (innovative or standard) were a priori forced into the model, as there were stratification covariates in CLEAN 3. Finally, we grouped the wrist and cubital fossa on one side, and the other three insertion sites on the other, as PIVC insertion at a joint site is more likely to result in PIVC dislodgment or dressing disruption, both factors increasing infectious risk. Analyses were performed using R 4.0.2 (R-project, Vienna, Austria) and *survival 3.5–7* package. A *p*-value equal to or lower than 0.05 was considered as significant.

## Results

Between Jan 7, 2019, and Sept 6, 2019, 1316 patients were eligible for the CLEAN 3 study and 1000 were enrolled. Of these, 177 PIVC were excluded for insertion failure (*n* = 6), consent withdrawal (*n* = 5), lack of PIVC tip culture (*n* = 143) and insertion site unknown (*n* = 23). Table 1 summarized the characteristics of the 823 patients and PIVC included in the current study. Catheter insertion was successful after the first, second, third and fourth attempt in 665, 112, 33 and 13 patients, respectively. Overall, 75 patients had PIVC colonization and/or local infection, and 173 PIVC tip culture were positive. Incidence of PIVC colonization and/or local infection and incidence of positive PIVC tip culture were not influenced by the number of puncture attempts (Tables S1 and S2). Tables S1 and S2 provide univariate analyses to identify covariates associated with PIVC colonization and/or local infection or with positive PIVC tip culture, respectively. Using adjusted multivariate Cox models and compared with forearm, PIVC insertion at the cubital fossa or wrist increased the risk of PIVC colonization and/or local infection (HR [95% CI], 1.64 [0.92—2.93] and 2.11 [1.08—4.13]) and of positive PIVC tip culture (1.49 [1.02—2.18] and 1.59 [0.98—2.59]), respectively (Table 2). After pooling insertion sites into two groups, PIVC insertion at a joint (wrist or cubital fossa) rather than another upper limb site increased the risk of PIVC colonization and/or local infection (HR [95% CI], 1.72 [1.08–2.75], *p* = 0.023) and of positive PIVC tip culture (HR [95% CI], 1.78 [0.98–1.81], *p* = 0.065).

**Table 1:** Patients and catheters chracteristics

	**Hand**		**Wrist**		**Forearm**		**Cubital fossa**		**Upper arm**	
	123	(15)	103	(12)	321	(38)	255	(30)	21	(2)
Gender, male	51	(41)	52	(50)	178	(55)	120	(47)	18	(86)
Age, years	75	[65–86]	82	[64–88]	79	[64–87]	72	[63–85]	75	[70–87]
Body mass index, kg/m^2^	27	[23–31]	26	[22–30]	24	[22–27]	25	[23–29]	25	[23–28]
Antiseptic group										
2% chlorhexidine-alcohol	70	(57)	44	(43)	158	(49)	139	(55)	9	(43)
5% povidone iodine-alcohol	53	(43)	59	(57)	163	(51)	116	(45)	12	(57)
Devices group										
Standard	67	(54)	55	(53)	152	(47)	110	(43)	12	(57)
Innovative	56	(46)	48	(47)	169	(53)	144	(56)	9	(43)
Chronic disease^a^										
Diabetes	39	(32)	17	(17)	62	(19)	43	(17)	6	(29)
Dyslipidemia	26	(21)	11	(11)	71	(22)	53	(21)	4	(19)
COPD	16	(13)	9	(9)	31	(10)	23	(9)	5	(24)
Chronic heart failure	20	(16)	24	(23)	57	(18)	36	(14)	6	(29)
Chronic renal failure	9	(7)	4	(4)	22	(7)	13	(5)	2	(10)
Long-term corticosteroids	2	(2)	5	(5)	14	(4)	5	(2)	0	(0)
Immune deficiency	4	(3)	0	(0)	7	(2)	3	(1)	1	(0)
Haematological malignancy	2	(2)	5	(5)	10	(3)	4	(2)	0	(0)
Autoimmune disease	4	(3)	1	(1)	12	(4)	11	(4)	0	(0)
Unknown	20	(16)	19	(18)	43	(13)	34	(13)	6	(29)
None	30	(24)	37	(36)	103	(32)	105	(41)	3	(14)
Antibiotics in the last 15 days	7	(6)	11	(11)	29	(9)	22	(9)	0	(0)
Number of catheter insertion attempts	1	[1–2]	1	[1–1]	1	[1–1]	1	[1–1]	1	[1–2]
Time with catheter in place, hours	43	[24–66]	39	[23–70]	42	[20–67]	32	[17–58]	47	[21–66]
Catheter colonization and/or local infection	9	(7)	16	(16)	21	(7)	28	(11)	1	(5)
Positive catheter tip culture	28	(23)	27	(26)	54	(17)	58	(23)	6	(29)

Data are n (%) or median [IQR]

*COPD* Chronic Obstructive Pulmonary Disease

^a^Some patients may have more than one chronic disease

**Table 2:** Adjusted hazard ratio by different insertion sites for catheter colonization and/or local infection, and for positive catheter culture, using multivariate Cox models

**Colonization and/or local infection 75/823**					
			Hazard Ratio	95% CI	*p* value
Insertion site					
	*Forearm*	21 (7)	-	-	-
	*Hand*	9 (7)	1.22	[0.55—2.69]	0.6
	*Upper arm*	1 (5)	0.63	[0.08—4.71]	0.7
	*Cubital fossa*	28 (11)	1.64	[0.92—2.93]	0.091
	*Wrist*	16 (16)	2.11	[1.08—4.13]	**0.030**
**Positive catheter culture 173/823**					
			Hazard Ratio	95% CI	*p* value
Insertion site					
	*Forearm*	54 (17)	-	-	-
	*Hand*	28 (23)	1.43	[0.89—2.29]	0.14
	*Upper arm*	6 (29)	1.81	[0.77—4.25]	0.2
	*Cubital fossa*	58 (23)	1.49	[1.02—2.18]	**0.038**
	*Wrist*	27 (26)	1.59	[0.98—2.59]	0.061

Data are n/N or n (%)

*CI* Confidence Interval

## Discussion

We carried out a post hoc analysis of CLEAN 3 database to assess the link between PIVC insertion site and its infectious risk. The value of the CLEAN 3 database is that it is recent and includes almost 1000 PIVCs with few missing data. Moreover, we used research staff to ensure high quality data collection and we sent over 85% of PIVC tips to the laboratory for culture. We used catheter colonization instead of PIVC-related BSI as it is by far a much more common event and is regularly used as a surrogate of PIVC-related BSI because colonization usually precedes BSI [8]. Using PIVC-related BSI would have required inclusion of tens of thousands of PIVC, which is difficult to achieve with the collection of large amounts of data and the sending of PIVC tips for culture.

In our study, insertion of the PIVCs at the wrist or cubital fossa increased the risk of colonization and/or local infection, as well as the risk of positive PIVC tip culture. The choice of the best PIVC insertion site provided conflicting results in the literature. In a secondary analysis involving 12 prospective studies and almost 12,000 PIVCs, insertion of PIVCs at the wrist or cubital fossa were associated with increased non-infectious complications leading to PIVC failure (i.e., infiltration, occlusion and dislodgment) [6]. Unfortunately, the association between PIVC insertion site and the risk of infectious complications were not investigated. In a retrospective study of 24 cases of PIVC-related BSI in adult patients, PIVC involved were more frequently inserted in the forearm and arm and less frequently inserted in the back of hand [5]. As this was a retrospective study, the authors included only cases for which all diagnostic criteria were met. Thus, the actual number of cases was probably underestimated, which could have influenced the impact of insertion site choice on PIVC-related BSI occurrence. In addition, the insertion sites for catheters without BSI were not recorded. Thus, it was not possible to establish a link between insertion site and infectious risk. Finally, in a large prospective cohort study involving more than 400,000 PIVC, hand insertion reduced the risk of PIVC-related BSI (HR [95% CI], 0.42 [0.18–0.98], *p* = 0.046) compared with proximal insertion sites [9].

We believe that insertion sites close to the joints could lead to PIVC dislodgment, thus damaging the endothelium of the vein and enabling bacteria from the insertion site to penetrate the body. These two components increase the risk of phlebitis and infectious as well as noninfectious complications. Moreover, the joints compromise the hold of the polyurethane dressing. Dressing disruption is a well-known major risk factor of infectious complications associated with vascular catheters [10].

In our study, the number of attempts for PIVC placement did not increase the risk of colonization and/or local infection, as well as the risk of positive PIVC tip culture. Few studies have examined this issue. A multicenter observational study of 5,300 PIVCs reported that more than two puncture attempts increased the number of catheter failures (HR [95% CI], 1.48 [1.19–1.84], *p* < 0.001), although this study did not specifically look at infectious complications. We believe that unlike with central venous catheters, where the same site is frequently punctured in the event of insertion failure, PIVC insertion failure requires the operator to change the insertion site, which may explain why the number of attempts is not correlated with risk of infectious complications.

Our study has several limitations. Firstly, this is a *post-hoc* analysis of a single-center study, which may compromise the external validity of the results. However, the large number of patients included and the wide range of medical conditions presented makes it possible to explore a representative sample of the general population. Secondly, only patients visiting our emergency department were included. PIVC inserted in emergency departments are at greater risk of infectious complications. However, only experienced nurses took part in the study, guidelines to prevent PIVC-related BSI were rigorously applied and PIVC inserted urgently were excluded. Thirdly, the study was not randomized according to insertion site. In emergency departments, PIVCs are mostly inserted in the cubital fossa or forearm, as these veins are easy to puncture and of large diameter. This enables insertion of larger-diameter PIVCs, which are more effective when vascular filling, blood products or contrast media administration are required. However, we did multivariate analyses taking into account all covariates of interest to identify independent factors associated with PIVC-related infectious complications.

The choice of the insertion site for a PIVC depends on a variety of factors, including the quality of the patient's venous network, the diameter of the catheter to be inserted, patient comfort and the risk of infectious and non-infectious complications. Our study suggests that the wrist and cubital fossa should be avoided whenever possible to reduce the risk of infectious complications. Prevention measures should consider the insertion site to reduce the risk of severe infections associated with PIVC.

### Supplementary Information

Supplementary Material 1.

Supplementary Material 2.

## Data Availability

The CLEAN 3 database is available on the Poitiers University Hospital statistical platform. CLEAN 3 database has not been published. All data generated or analysed during this study are included in this published article and its supplementary information files.

## Abbreviations

BSI: Bloodstream Infection
CFU: Colony Forming Units
HR: Hazard Ratio
IQR: Interquartile Range
PIVC: Peripheral Intravenous Catheter
