# Supplementary material for: Insertion site and risk of peripheral intravenous catheter colonization and/or local infection: a post hoc analysis of the CLEAN 3 study including more than 800 catheters
Source: Antimicrob Resist Infect Control. 2024 Jun 5;13:57. doi: 10.1186/s13756-024-01414-4 (PMC11151591; doi:10.1186/s13756-024-01414-4)
Supplement: Supplementary file 1 — Supplementary Material 1. [file 13756_2024_1414_MOESM1_ESM.docx]

**Supplementary file 1. Univariate analysis for catheter colonization and/or local infection**

| **Characteristic** | **No colonization and/or no local infection** | **Colonization and/ or local infection** | **p-value** |
| --- | --- | --- | --- |
|  | n=748 | n=75 |  |
| Body mass index, kg/m^2^ | 24.9 [22.2-29.3] | 24.8 [22.1-29.2] | 0.5 |
| Smoker | 101 (13) | 11 (15) | 0.7 |
| Chronic disease* |  |  |  |
| Diabete | 153 (20) | 14 (19) | 0.8 |
| Dyslipidemia | 149 (20) | 16 (22) | 0.7 |
| COPD | 78 (10) | 7 (8) | 0.6 |
| Chronic heart failure | 128 (17) | 15 (21) | 0.5 |
| Chronic renal failure | 41 (6) | 9 (12) | **0.034** |
| Long-term corticosteroïds | 26 (4) | 0 (0) | 0.2 |
| Immune deficiency | 13 (2) | 1 (1) | >0.9 |
| Haematological malignancy | 19 (3) | 2 (3) | 0.7 |
| Autoimmune disease | 25 (3) | 3 (4) | 0.7 |
| Antibiotics in the last 15 days | 37 (5) | 3 (4) | >0.9 |
| Antiseptic group |  |  | **<0.001** |
| 2% chlorhexidine-alcohol | 415 (55) | 4 (5) |  |
| 5% povidone iodine-alcohol | 333 (45) | 71 (95) |  |
| Devices group |  |  | 0.4 |
| Innovative | 386 (52) | 41 (56) |  |
| Standard | 362 (48) | 32 (44) |  |
| Skin shaving | 2 (0) | 0 (0) | >0.9 |
| Insertion site |  |  | **0.046** |
| Forearm | 300 (40) | 21 (27) |  |
| Hand | 114 (15) | 9 (12) |  |
| Upper arm | 20 (3) | 1 (1) |  |
| Cubital fossa | 227 (30) | 28 (37) |  |
| Wrist | 87 (12) | 16 (22) |  |
| Catheter size (Gauge) |  |  | 0.7 |
| 16 | 1 (0) | 0 (0) |  |
| 18 | 521 (70) | 55 (74) |  |
| 20 | 215 (29) | 20 (26) |  |
| 22 | 11 (2) | 0 (0) |  |
| Insertion attempt |  |  | 0.9 |
| 1 | 602 (80) | 63 (84) |  |
| 2 | 104 (14) | 8 (11) |  |
| 3 | 30 (4) | 3 (4) |  |
| 4 | 8 (1) | 1 (1) |  |
| >4 | 4 (1) | 0 (0) |  |
| Time with catheter in place, h | 39 [20-62] | 69 [46-113] | **<0.001** |

Data are n (%) or median [IQR]. COPD = Chronic Obstructive Pulmonary Disease. *Some patients may have more than one chronic disease
