# Supplementary material for: Insertion site and risk of peripheral intravenous catheter colonization and/or local infection: a post hoc analysis of the CLEAN 3 study including more than 800 catheters
Source: Antimicrob Resist Infect Control. 2024 Jun 5;13:57. doi: 10.1186/s13756-024-01414-4 (PMC11151591; doi:10.1186/s13756-024-01414-4)
Supplement: Supplementary file 2 — Supplementary Material 2. [file 13756_2024_1414_MOESM2_ESM.docx]

**Supplementary file 2. Univariate analysis for positive catheter tips culture.**

| **Characteristic** | **Sterile culture** | **Positive culture** | **p-value** |
| --- | --- | --- | --- |
|  | n=650 | n=173 |  |
| Body Mass Index, kg/m^2^ | 24.9 [22.3-29.3] | 24.6 [22.0-29.1] | 0.4 |
| Smoker | 86 (13) | 26 (15) | 0.5 |
| Chronic disease* |  |  |  |
| Diabete | 123 (19) | 44 (25) | 0.058 |
| Dyslipidemia | 123 (19) | 42 (24) | 0.12 |
| COPD | 69 (11) | 15 (9) | 0.5 |
| Chronic heart failure | 106 (16) | 37 (21) | 0.12 |
| Chronic renal failure | 33 (5) | 17 (10) | **0 .020** |
| Long-term corticosteroïds | 22 (3) | 4 (2) | 0.5 |
| Immune deficiency | 9 (1) | 5 (3) | 0.2 |
| Haematological malignancy | 12 (2) | 9 (5) | **0.025** |
| Autoimmune disease | 22 (3) | 6 (3) | >0.9 |
| Antibiotics in the last 15 days | 32 (5) | 8 (5) | 0.9 |
| Antiseptic group |  |  | **<0.001** |
| 2% chlorhexidine-alcohol | 376 (58) | 44 (25) |  |
| 5% povidone iodine-alcohol iodineiodine | 274 (42) | 129 (75) |  |
| Devices group |  |  | 0.8 |
| Innovative | 336 (52) | 91 (52) |  |
| Standard | 314 (48) | 82 (47) |  |
| Skin shaving | 2 (0) | 0 (0) | >0.9 |
| Insertion site |  |  | 0.14 |
| Forearm | 267 (41) | 54 (31) |  |
| Hand | 95 (15) | 28 (16) |  |
| Upper arm | 15 (2) | 6 (4) |  |
| Cubital fossa | 197 (30) | 58 (34) |  |
| Wrist | 76 (12) | 27 (16) |  |
| Unknown | 17 | 6 |  |
| Catheter size (Gauge) |  |  | 0.3 |
| 16 | 0 (0) | 1 (1) |  |
| 18 | 451 (69) | 125 (72) |  |
| 20 | 190 (29) | 45 (26) |  |
| 22 | 9 (1) | 2 (1) |  |
| No. of insertion attempt |  |  | >0.9 |
| 1 | 524 (81) | 141 (82) |  |
| 2 | 90 (14) | 22 (13) |  |
| 3 | 25 (4) | 8 (5) |  |
| 4 | 8 (1) | 1 (1) |  |
| >4 | 3 (1) | 1 (1) |  |
| Time with catheter in place, h | 37 [20-58] | 54 [36-88] | **<0.001** |
|  |  |  |  |

Data are n (%) or median [IQR]. COPD = Chronic Obstructive Pulmonary Disease. *Some patients may have more than one chronic disease
